# Effects of Molecular Chain Length on the Contact Line Movement in Water/*n*-Alkane/Solid Systems

**DOI:** 10.3390/polym11122081

**Published:** 2019-12-12

**Authors:** Wenxiu Zheng, Chengzhen Sun, Boyao Wen, Bofeng Bai, Eric Lichtfouse

**Affiliations:** State Key Laboratory of Multiphase Flow in Power Engineering, Xi’an Jiaotong University, Xi’an 710049, China; zhengwenxiu1989@stu.xjtu.edu.cn (W.Z.); sun-cz@xjtu.edu.cn (C.S.); wbyo721@stu.xjtu.edu.cn (B.W.); eric.lichtfouse@inra.fr (E.L.)

**Keywords:** molecular chain length, contact line, wettability, liquid-liquid-solid system

## Abstract

The movement of the contact line in liquid-liquid-solid systems is a major phenomenon in natural and industrial processes. In particular, *n*-alkanes are widely occurring in the oil, soil pollution, and chemical industries, yet there is little knowledge on the effects of molecular chain length on the contact line movement. Here, we studied the effects of molecular chain length on the contact line movement in water/*n*-alkane/solid systems with different surface wettabilities. We used *n*-heptane (C_7_), *n*-decane (C_10_), and *n*-hexadecane (C_16_) as alkanes and *α*-quartz as the solid surface. We calculated the time-variation contact line moving velocity and also analyzed the jump frequency and the mean distance of the molecular displacement occurring within the contact line zone by molecular-kinetic theory. Molecular dynamics simulation results show that the contact line velocity decreases with increasing the chain length, originally caused by the decreasing the jump frequency and mean distance. These variations with the molecular chain length are related to the more torsions and deformations of the molecules with a longer chain length. In addition, the moving mechanism of the contact line on the same solid surface does not change at different molecular chain lengths, implying that the moving mechanism mainly depends on the three-phase wettability.

## 1. Introduction

The phenomenon of water displacing oil occurs in many processes such as dish and cloth washing and petroleum recovery [1], where the three-phase contact line plays significant role. Research is thus focusing on the three-phase contact line in the liquid-liquid-solid systems [2,3,4,5]. In particular, understanding of the three-phase contact line behaviors on the molecular level is necessary since the contact line occurs at where the liquid-liquid/vapor interface meets the solid surfaces [6,7,8,9]. Indeed, the viscosity and the high density of the molecules near the contact line can induce specific moving mechanisms [10]. In addition, the single-molecule adsorption-desorption mechanism of the liquid-vapor-solid system is not suitable for the liquid-liquid-solid systems [11,12,13]. We showed previously that the moving mechanisms of the three-phase contact line in a liquid-liquid-solid system were greatly related to the three-phase wettability. In systems with different mechanisms, the trends of the time-variation velocity of the contact line and the parameters of the molecular-kinetics theory showed great differences [14].

The *n*-alkanes are widely occurring in the oil, soil pollution, and chemical industries [15,16]. *n*-Alkane molecules have flexible chains that may be oriented [17] under solid-liquid interactions, thus affecting the movement of the contact line [18]. The contact line movement is also likely to be controlled by the torsions of *n*-alkane chains [19]. In industrial processes such as oil recovery, *n*-alkanes are presented as the mixtures of homologues with various molecular lengths. Therefore, studying the effects of molecular chain length on the contact line movement is crucial. Suggestions in regulating and controlling the contact line movement can be given from the perspective of changing the parameters of the molecular-kinetics theory in systems with different molecular chain length. Previous investigations have evidenced the parallel orientation of alkane chains [17] and the effect of chain length on the contact line friction [18,20]. However, little knowledge is drawn on the effects of the chain length on the contact line movement from molecular level and whether it will make a transformation of the moving mechanism from one to another. Until now, this issue has not been examined systematically. 

Therefore, we study here the effects of chain length on the contact line movement at a liquid-liquid-solid system with different surface wettabilities. The molecular dynamics simulation method is adopted, which is a widely used method capturing the movements of the atomic particles controlled by Newton’s laws of motion and is used in many applications [21,22,23,24]. We detail the molecular dynamics model and give the results of the distributions of the liquid molecules near the solid surface and the time-variation velocity of the contact line. We also calculate the parameters used in the molecular-kinetics theory.

## 2. Model and Methods

**Figure 1:**
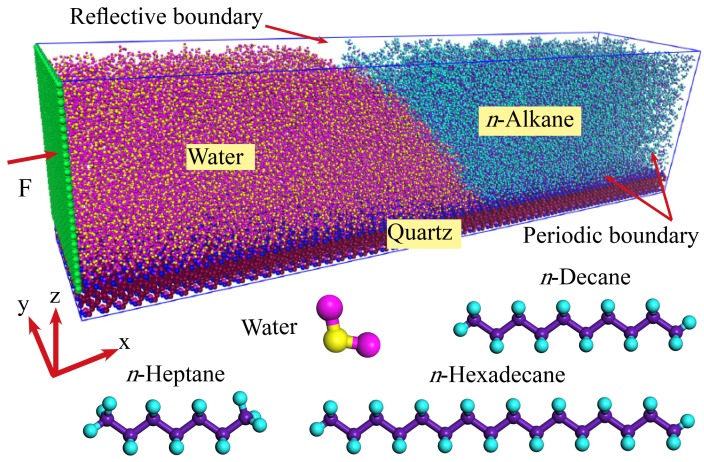
Simulation model of the displacing process in the water/*n*-alkane/quartz systems. Structures of water and *n*-alkanes, heptane (C_7_), decane (C_10_), and hexadecane (C_16_) are given.

Molecular dynamics simulations are implemented in the water/*n*-alkane/solid three-phase systems using the LAMMPS software. The two liquids are placed on a 28.4 nm × 6 nm *α*-quartz surface, as shown in Figure 1. The surface is smooth with no roughness. *n*-Heptane (C_7_), *n*-decane (C_10_), and *n*-hexadecane (C_16_) are used as the alkane phase. The displacing side contains 16,245 molecules of H_2_O with transferable intermolecular potential with 3 points (TIP3P) model. Simulations are performed using periodic boundary conditions in the directions parallel to the silica surface, and using reflective boundary conditions in the directions vertical to the silica surface [14]. For the *α*-quartz surface, the non-bridging oxygen atoms which are bonded to only one Si atom are attached to the hydrogen atoms [25]. The surface atoms are fixed in the simulations to avoid the weak surface vibrations resulting from collisions with *n*-alkane and H_2_O molecules. This allows us to better assess the morphology of the molecules near the three-phase contact lines.

The simulations are firstly run for enough time to reach the equilibrium in the canonical (NVT) ensemble. Then a 6 nm × 6.4 nm wall is added from the left of the displacing side to drive the movement (Figure 1). We previously described the simulation procedures [14]. An external force *F* is added to drive the systems, then simulations are performed to reach a steady state in the systems. In the simulations, the time step is set to 1.0 fs and the moving process of the three-phase contact line is recorded with a period of 10,000 steps (0.01 ns). The system temperature is of 300 K in the NVT ensemble for different *n*-alkane systems.

We adopt several models to simulate the interactions among atoms. For the added wall, we applied the adaptive intermolecular reactive empirical bond-order (AIREBO) potential model, whose form and parameters are given by Stuart et al. [26]. We used the Lennard-Jones (L-J) potential model coupling with the Coulombic potential for the liquid and *α*-quartz atoms. Here, the cutoff radius (*r*_cut_) is set at 1.0 nm, which is acceptable and widely used [27]. The long-range Coulombic interactions are taken into account using the particle-particle particle-mesh (PPPM) method. The terms of the Lennard-Jones (L-J) potential (Equation (1)) and the Coulombic potential (Equation (2)) can be described as follows:

(1) $$\phi \left(r_{ij}\right)=4\varepsilon \left[{\left(\frac{\sigma }{r_{ij}}\right)}^{12}-{\left(\frac{\sigma }{r_{ij}}\right)}^{6}\right]$$

where *r_ij_* is the distance between atoms *i* and *j*, *ε* is the energy parameter and *σ* the length scale.

(2) $$\phi_{c}(r_{ij})=\frac{Qq_{i}q_{j}}{\varepsilon_{0}r_{ij}}$$

where *q_i_* and *q_j_* are the charges of atoms *i* and *j*, *Q* is the energy-conversion constant, *ε*_0_ is the dielectric constant in vacuo.

The interactions between *α*-quartz atoms and atoms in liquid molecules are modeled using the hybrid potential model. The L-J potential parameters between the crossing atoms are calculated using the Lorentz-Berthelot mixing rule. The charges of *n*-alkane atoms are calculated by the DMol^3^ module of Materials Studio 7.0. These potential parameters have been verified [21,22].

We have found that the moving contact line has several typical mechanisms at hydrophilic, intermediate, and hydrophobic systems. Noteworthy, in the hydrophobic system the contact line is hard to be driven [14]. Thus, here we only consider two typical three-phase wettabilities (hydrophilic/45° and intermediate/90°) by changing the energy parameter *ε* in Equation (1). For simplicity, the constant *E* enables us to selectively increase or decrease the interactions between different types of atoms by writing the energy parameter in Equation (1) as *E*·*ε*. We assign different types of interactions as *E*_water-solid_ and *E*_alkane-solid_ to represent the water-solid interactions and the *n*-alkane-solid interactions. In our simulations, the solid surface is both physically and chemically uniform. Therefore, the wettability provides a useful way to test the quality of the surface. Here, we consider the *E*_water-solid_ of 1 and 0.2, the *E*_alkane-solid_ is 1 in both cases, and the resulting three-phase wettability (contact angle of water side) is 45° and 90°, respectively. The equilibrium contact angles are obtained by linear fitting of the *n*-alkane/water interface points [28]. Since the parameters in the potential models are the same for the C_7_, C_10_ and C_16_, the wettabilities are almost the same in different chain length systems; thus, we can neglect the small differences.

## 3. Results and Discussion

### 3.1. Density of Water and Alkane Molecules Near the Solid Surface

The characteristics of the liquid molecules near the solid surfaces are crucial in understanding the contact line movement. In particular, liquid molecules can be adsorbed onto the solid surface to form a high-density zone. Here we obtained the molecular density distribution along the *z*-direction by dividing the simulation regions into bins. The bin size Δ*z* was *σ*/5 [29]. Here *σ* is the length scale in Equation (1). We use the oxygen atoms to represent the water molecules because one water molecule has only one oxygen atom. Similarly, the carbon atoms are adopted to represent the alkane molecules. For oxygen atoms, the *σ* is 3.15 Å and for carbon atoms the *σ* is 3.50 Å. So the bins should be 0.63 Å and 0.70 Å, separately. *z* coordinates of H_2_O and *n*-alkane molecules are stored in bins. Then we calculated the number density distribution based on Equation (3):

(3) D=n(b)/S

where *n*(*b*) represents the number of elements in the *b*th bin and *S* is the projected area of the elements on the *x-y* plane.

**Figure 2:**
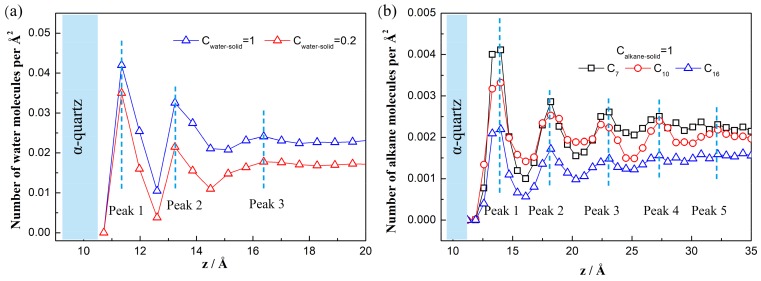
Number density near a quartz surface along the *z*-coordinates. (**a**) Water molecules. C is the liquid-solid energy coefficient. Blue lines represent a contact angle of 45° and red lines a contact angle of 90°. (**b**) n-Alkane molecules.

Figure 2a shows the number density distribution near the substrate along the direction perpendicular to the solid surface. It is found that the water peaks appear at *z* = 11.34 Å, *z* = 13.23 Å, and *z* = 16.39 Å, corresponding respectively to the distance 0.14 nm, 0.33 nm, and 0.65 nm away from the O-surface. This finding is in agreement with the results from Emami et al. [30]. The first peak is caused by the solid hydroxyl groups that form H-bond with water molecules [31]. The second and third peaks are also explained by strong water-solid interactions, including the Van der Waals force and the electronic force. Additionally, we also observe that the stronger solid-water interactions (larger C) exhibit more intense peaks, which indicate a stronger adsorption force of the solid surfaces to the water molecules.

For the *n*-alkane molecules, we find that there are several peaks for the *n*-alkane adsorption, as shown in Figure 2b. The peaks are generated by the strong *n*-alkane/solid interactions. In addition, the chain-like structure of *n*-alkane is likely to modify molecular distribution in a confined space, particularly at the nano-scale level [32]. When the peak is farther from the solid surface, the intensity is weaker due to the weaker interaction between the *n*-alkane molecules and the solid surface. For the bulk phase out of the layers, the density distribution is nearly uniform. Furthermore, for a longer chain length, the number of alkane molecules per Å^2^ is smaller at the same *z* coordinate since the molecular volume is larger.

### 3.2. Tripping Force

**Figure 3:**
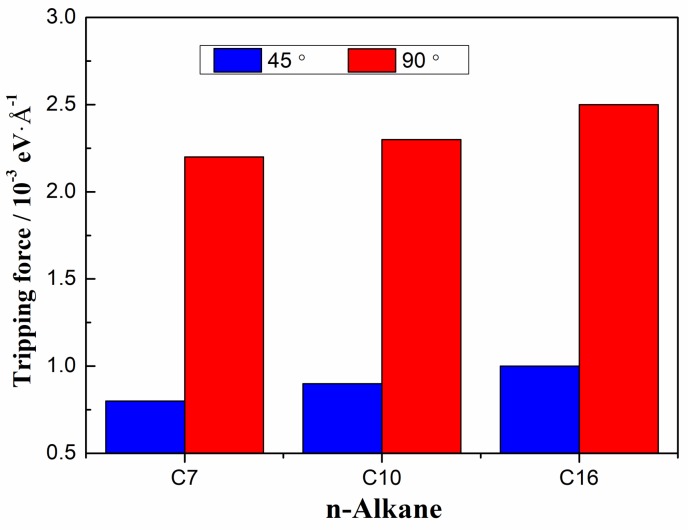
Tripping forces of water/*n*-alkane/solid systems.

The three-phase contact line is driven by an external driving force *F*. If *F* is too low, the system is static. If *F* is too high, a steady velocity cannot be reached by the simulation due to continuous interfacial deformations till the contact line disappeared. So a reasonable *F* is crucial to allow the contact line and water/*n*-alkane interface to reach a stable state, in which cases the contact line can achieve a steady velocity. Here, we defined a tripping force, which is the minimum force moving the contact line to reach a steady state. We firstly study the tripping forces in different systems, which are necessary to the first step of the contact line movement. Figure 3 shows the effects of alkane chain length on the tripping force. Results show that the tripping force highly increases with decreasing the hydrophilicity due to the weaker water-solid interaction. We observe that the tripping force increases with the chain length. This finding is probably due to the fact that longer chains have more torsions, together with the induced entangled ‘spaghetti-like’ balls by the neighboring molecules, causing a larger resistance.

### 3.3. Time-Averaged Velocity

**Figure 4:**
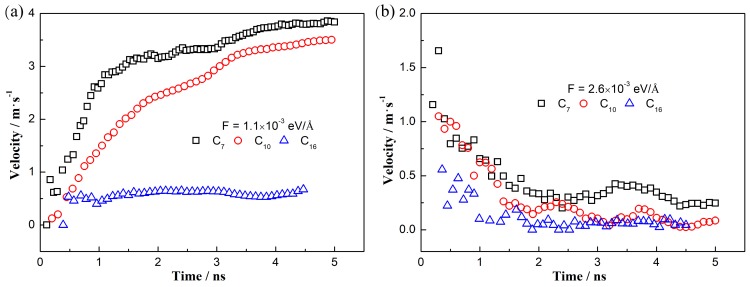
Contact line velocity versus time for the water/*n*-alkane/solid systems with different molecular chain length. (**a**) Wettability of 45°. (**b**) Wettability of 90°.

We analyzed the velocities of the three-phase contact line. The coordinates of the three-phase contact line at different times can be obtained by intersecting the fitting line of the water/*n*-alkane interface points and the solid surfaces. Then we use these coordinates to calculate the displacements of the contact line. The average velocity is used because of the weak fluctuation in the velocity caused by the thermal effects of the molecules. The effects of chain length, from C_7_ to C_16_, on the time-variation of the velocity are shown in Figure 4.

The contact line velocities in the hydrophilic cases (wettability of 45°) are shown in Figure 4a. A driving force of 1.1 × 10^−3^ eV/Å is adopted to obtain the effects of the molecular chain length. Here, the time-variation velocity decreases with increasing the molecular chain length. However, the trend that the velocity increases firstly and then reaches to a steady value (with weak fluctuation due to the molecular movement at nano-scale) is almost the same at different chain length, indicating that the moving mechanism of “Roll up” is the same at all of these cases [14].

For the intermediate cases (wettability of 90°), the results of the contact line velocities are shown in Figure 4b. The effects of molecular chain length are studied by using a driving force of 2.6 × 10^−3^ eV/Å. Similarly, we observe that the time-variation velocity decreases with increasing the molecular chain length. The trend that the contact line velocity has a maximum value at the start-up stage and then decreases to a steady value (with weak fluctuation due to the molecular movement at nano-scale) is almost the same at different chain length, indicating the same moving mechanism of “Piston” at all of these cases [14].

To further explain these phenomena, we analyze the driving force and the resistant force of the three-phase contact line at different chain length. The driving force in all systems is the unbalanced Young force generated by the external force [10]. The resistant force depends on the properties of the two liquids and the solid surfaces. Hence, for a system with a longer chain length, more dihedrals results in the more torsions and deformations at nano-scale, inducing a larger resistance and slow the contact line velocity. Consequently, from this evidence we can know that a longer chain length will slow the time-variation velocity of the contact line. However, the molecular chain length will not affect the moving mechanism of the contact line, implying that the moving mechanism depends on the three-phase wettability.

### 3.4. Parameters of Molecular-Kinetics Theory

In order to further explain the effects from a quantitative perspective, we try to obtain the parameters of molecular-kinetics theory at different molecular chain lengths. The molecular-kinetics theory [33] can describe the relationship between the contact line velocity *v* and the dynamic contact angle *θ_d_* [11], as given in Equation (4),

(4) $$v=2K_{0}\lambda \sinh\left[\frac{\lambda^{2}\gamma_{L_{1}L_{2}}}{2k_{B}T}(\cos\theta_{0}-\cos\theta_{d})\right]$$

where *k_B_* and *T* are the Boltzmann constant and the absolute temperature, respectively. $\gamma_{L_{1}L_{2}}$ is the water/*n*-alkane interfacial tension (49.62 mN/m, 44.34 mN/m, and 47.5 mN/m for C_7_, C_10_, and C_16_ systems, separately, theoretically obtained by the method in our group [22]), and*θ*_0_ is the equilibrium three-phase contact angle of the displacing side (water side, smaller with stronger water-solid interactions). *K*_0_ and *λ* represent the jump frequency and the mean distance of the molecular displacement occurring within contact line zone, respectively.

**Figure 5:**
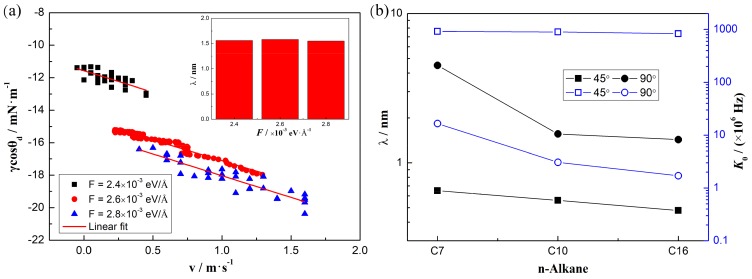
Jump mean distance λ and frequency *K*_0_ of the molecular displacement occurring within contact line zone, obtained by the molecular-kinetics theory. (**a**) The case of water/n-decane/solid system with the wettability of 90°: linear fitting of the molecular-kinetics theory and the jump mean distance under different driving forces. (**b**) The black one represents the mean distance and the blue one represents the frequency.

In this work, we adopt the method by Renate Fetzer et al. [34] to obtain the two parameters, which is also verified in our previous work [14]. *K*_0_ and *λ* are directly obtained by reducing Equation (4) to a single exponential form, results are shown in Figure 5. The *K*_0_ and *λ* are the basic characteristics of the system and are affected by the properties of the liquids and the substrate, which have nothing to do with the driving forces. An example of water/n-decane/solid system with the wettability of 90° is shown in Figure 5a. We can see that the slope of the fitting line is almost the same with increasing the driving force, meaning the same contact line friction [10] and reflecting the unchanged mean distance (about 1.56 nm) under different driving forces. The mean distance *λ* in Figure 5b increases with decreasing the hydrophilicity and the value is on the same order of magnitude as other works [11,12,35]. However, with decreasing the hydrophilicity, the frequency *K*_0_ changes for about 2 orders of magnitude, which verified the different mechanisms at different surface wettabilities [14]. The mean distance *λ* decreases as the molecular chain length increases; namely, from 0.65 nm to 0.48 nm at the wettability of 45°, and from 4.5 nm to 1.43 nm at the wettability of 90°. Additionally, the frequency *K*_0_ will also decrease with increasing the molecular chain length; from 918 × 10^6^ Hz to 835 × 10^6^ Hz at the wettability of 45°, and from 16.55 × 10^6^ Hz to 1.7 × 10^6^ Hz at the wettability of 90°. These decreases are resulted from the more torsions and deformations of the molecules at the nano-scale. The lower frequency and mean distance imply a larger resistance and show us why the velocity is slower at a longer molecular chain system. These results give us more physical explanations about the effects of the molecular chain length on the contact line movement in a liquid-liquid-solid system.

## 4. Conclusions

Surfaces with different wettabilities are adopted to study the effects of the molecular chain length on the contact line movement in water/*n*-alkane/solid systems via molecular dynamics simulations. The time-variation contact line velocities are calculated and the key parameters (jump frequency and the mean distance) obtained by the molecular-kinetics theory are analyzed to get a deep understanding of the contact line movement from the physical viewpoint. Results show that a longer chain length makes the contact line harder to move and increases the tripping force, and that the time-variation contact line velocity decreases at the same driving force. In addition, a longer molecular chain length decreases the jump frequency and the mean distance owing to the more torsions and deformations, resulting in a larger contact line friction and therefore slows the contact line movement. However, the molecular chain length will not affect the moving mechanism of the contact line, implying that the moving mechanism mainly depends on the three-phase wettability. This work is helpful to give suggestions in regulating and controlling the contact line movement in systems with different molecular chain length.
